# Epidemiology of nodding syndrome in the Greater Mundri area, South Sudan: Prevalence, spatial pattern and environmental risk factors

**DOI:** 10.1371/journal.pntd.0010630

**Published:** 2022-07-28

**Authors:** Gasim Omer Elkhalifa Abd-Elfarag, Lukudu Emmanuel, Arthur W. D. Edridge, Stella van Beers, Mohamed B. Sebit, Michaël B. van Hensbroek, Ente J. J. Rood

**Affiliations:** 1 Amsterdam Center for Global Health, Department of Pediatrics and Department of Global Health, Amsterdam UMC, Amsterdam, the Netherlands; 2 Access for Humanity (AFH), Monitoring and Evaluation Unit, Juba, the Republic of South Sudan; 3 Laboratory of Experimental Virology, Department of Medical Microbiology, Amsterdam UMC, Amsterdam, the Netherlands; 4 Kit-Royal Tropical Institute, Epidemiology, Center for Applied Spatial Epidemiology (CASE), Amsterdam, the Netherlands; 5 Department of Psychiatry, College of Medicine, University of Juba, Juba, the Republic of South Sudan; Imperial College London, Faculty of Medicine, School of Public Health, UNITED KINGDOM

## Abstract

**Background:**

Nodding syndrome (NS) is a progressive neurological disease that has been described in several sub-Saharan African counties, but South Sudan is considered the most affected. However, knowledge about the exact burden and the epidemiological risk factors of NS in South Sudan is lacking.

**Objective:**

To determine the prevalence, distribution and epidemiological risk factors of NS in the Greater Mundri area, the epicenter of NS in South Sudan.

**Methods:**

A NS prevalence house-to-house survey was conducted in multiple villages between February 2018 and November 2019. Geographical distribution and clustering of NS cases was identified using spatial and binomial regression analysis. Epidemiological risk factors of NS were identified using univariate and multivariate models.

**Results:**

Of the 22,411 persons surveyed in 92 villages, 607 (2.7%) persons with NS were identified, of which 114 (19%) were new-onset cases. The highest prevalence was found in Diko village with a prevalence of 13.7%. NS showed a significant spatial pattern with clustering of cases between adjacent households and along rivers. Risks factors for NS include all behaviors around rivers (drinking, cooking, handwashing and bathing) and exposure to poultry. On the other hand, ownership of mobile phone decreased the risk of NS. Many other factors, including prior ivermectin treatment and internal displacement were not associated with NS.

**Conclusion:**

Our study demonstrates a very high burden of the NS disease in the Greater Mundri area, strengthens the association with rivers, and identified possible new clues for an underlying cause.

## Author summary

Nodding syndrome (NS) is a neglected and progressive neurological disorder that is disabling and of unknown cause. The Greater Mundri area in South Sudan is currently considered to be the epicenter of new cases. In this study, we evaluated the prevalence, spatial pattern and epidemiological risks of NS. We found that the prevalence of NS is very high–more than one in five households affected–in the Greater Mundri area, which is comparable with the previously reported rates within smaller populations in the same area, but higher than the rates reported in all the other NS-endemic countries. We found that NS cases significantly cluster between adjacent households but we could not find clustering within the same household. In addition, we prove that living near the rivers, as well as using river water for drinking, cooking, handwashing and bathing strongly increased the risk of having a NS case in households. We further identified a new association of NS with ownership of poultry.

## Introduction

Nodding syndrome (NS) is a progressive neurological disorder that has been first described in southern Tanzania in 1934 [1]. Several decades later, similar cases were also reported from restricted areas in Liberia, South Sudan and Uganda [1–3]. Recently, there are new reports from the Democratic Republic of Congo (DRC), Cameroon and the Central African Republic [4–6]. South Sudan is currently considered the most affected with hundreds of new cases that continue to emerge from the Greater Mundri area in Western Equatoria State [2]. Previous studies in the Greater Mundri area described a range of NS prevalence rates in smaller population within restricted areas in Lui and Amadi villages of 2.3% and 6.7%, respectively [7]. NS often presents with distinctive clinical feature of head nodding, followed by incapacitating complications, including generalized convulsions and severe mental retardation, which often may lead to premature death [1,2,8,9].

Infections with filarial parasites (*Onchocerca volvulus and Mansonella perstans)*, viruses (measles), nutritional deficiencies (vitamin B6) and exposure to toxins (mouldy maize, red/brown sorghum, traditional medicines, and chemicals from munition) have been hypothesized and studied as possible causes for NS. Most of these studies focused on *Onchocerca volvulus* (OV) and reported a significant association with NS. In addition, clustering of NS cases within families and near fast-flowing rivers were also reported [2,4,5,8,10–14].

However, to date, conclusive evidence regarding risk factors resulting in NS have not been identified. To better understand the etiology and develop and implement appropriate public health interventions to address the NS epidemic, a better understanding of the epidemiology, including the scale of the disease is urgently needed. We therefore embarked on a large scale, high resolution epidemiological assessment of NS burden and the epidemiological risks in the high endemic area of Western Equatoria state, the Greater Mundri.

## Methods

### Ethics statement

The study protocol was approved by the ethics committee of the Ministry of Health of the Republic of South Sudan. Thumb print or signed informed consent and assent was obtained from study participants or their parents or guardians. All study participants personal information were treated with strict confidentiality and coded for anonymity.

### Study setting

This study was conducted in 13 bomas in the Greater Mundri area, Western Equatoria state of South Sudan from February 2018 to November 2019. This area consists of 2 counties (Mundri West and Mundri East), which are sub-divided into 4 and 5 payams, respectively. Each payam is further sub-divided into an average of 4 bomas, consisting of several individual villages. Based on the 2008 South Sudan Population Census, the population of the Greater Mundri is estimated at 82,293. The 2020 projected population is estimated at 142,633 (46,760 Mundri West and 95,873 Mundri East) [15,16]. Subsistence farming is the main livelihood activity, with fishing and animal husbandry as additional livelihood activities. The area is undulating, flat, open woodland savannah with narrow streamlines and medium-length rainfall of 5 to 6 months. Water is mainly accessed from boreholes, streams and the Yei River. As a result of the civil conflict, which erupted in 2013, there has been significant population movement in and out of the area and between villages.

### Study design and procedures

This study formed part of a larger South Sudan NS research project which consist of: (1) an epidemiological study to determine the prevalence, distribution and risk factors of NS, (2) a clinical case-control study to determine the etiology of NS and (3) a follow-up study to investigate the disease progression and long-term outcome of NS. The epidemiological data was collected through a large household survey aiming to also identify the cases and controls for the etiology study.

Prior to initiating the household survey, community leaders in the area were informed about the aims and procedures of the study and involved in the planning and preparation of the study implementation in their communities. The study was executed by a team of researchers consisting of a medical doctor, a clinical officer, nurses, field workers and a field coordinator, who were trained prior to the survey.

The survey was implemented in three stages. Firstly, following informed consent, the main caretaker was interviewed and all persons in the household were screened for NS based on the clinical case definition [Table 1]. The case definition was formulated based on existing data (The 2012 consensus case definition for NS, International Scientific Meeting in Kampala, Uganda) and expert consolation workshop organized in Amsterdam in 2014, which included key medical professionals and NS experts [17]. Secondly, if children eligible to participate in the clinical case-control study were identified, additional questions on the child’s behavior and living conditions were asked. Thirdly, every fifth household and each household with eligible case or control were asked additional questions regarding their household circumstances, resources and socioeconomic status. Cases and controls were matched for age and sex. Household interviews were performed using electronic questionnaires preloaded on a personal digital assistant (PDA)/tablet. The tablets (Samsung) were used by the study team to also obtain geocoordinates of all households visited. In addition, a paper-based household listing form was used by the study team to enumerate households visited and record household size and the presence of suspect NS cases. The questionnaires were translated into the local language and back translated into English to ensure consistency. All the research team members are from the Greater Mundri Area and are fluent in the local languages, including the local Arabic.

**Table 1:** Nodding syndrome case definition.

**Suspected**: Reported head nodding or repeated unprovoked convulsions in a previously healthy person.
**Probable**: Suspected case, with ‘head nodding’ (as major) and at least one minor criteria or with ‘repeated convulsions’ (as major) and at least 2 minor criteria. Major criteria: • Head nodding with a frequency of 5–20 times/min • History of repeated generalized convulsions Minor criteria: • Other neurologic abnormalities (cognitive/behavior, etc.) • Clustering in space or time with similar cases • Triggering by eating or cold weather • Stunting or wasting • Delayed sexual or physical development • Psychiatric manifestations
**Confirmed**: Probable case, with documented head nodding episodes: • Observed and recorded by a trained health care worker AND/OR confirmation of presence of criteria to define ‘probable case’ by MD or trained healthcare worker: • Videotaped head nodding episode • Video/EEG/EMG documenting head nodding as atonic seizures

### Data quality and analysis

After completion of household interviews, the field coordinator conducted data quality checks, including personnel identifier code correctness, signed informed consent forms for household interviews and enrollment as cases/controls, data for completeness and accuracy and corrected inconsistencies, and completed household listing forms. Once the data quality checks were completed, data were extracted and uploaded directly from the ODK collect kit to the KoBoToolbox online database server for later analysis.

Data were extracted from the online database and cleaned and de-duplicated before subsequent analysis. Field supervisory forms, collected using the paper-based household listing form were also digitized. Both databases were merged using the unique household identifier and used to cross validate the number of suspect NS cases per household. The prevalence of NS was calculated by dividing the total number of suspect cases by the total number of persons screened within the study area and per boma.

To assess whether NS cases are found in geographic clusters or hotspots, the occurrence of suspect NS cases at household locations were regressed against a spatially lagged dependent variable. Statistical significance of clustering was assessed by using a binomial regression including the distance weighted proportion of NS within the 5 nearest neighboring households around each household as a covariate.

The univariate and multivariate effects of risk factors on the probability that one or more children within a single household were affected by NS was assessed by fitting binomial generalized linear models with a logit link to the data. To ensure that the model was not affected by spatial clustering of the data, which is indicative of a violation of the assumption of independence, the regression residuals were tested for spatial autocorrelation. The absence of spatial dependency implies that any observed clustering was fully accounted for by the covariates in the model.

To assess the singular and combined risk of environmental and socioeconomic factors univariate and multivariate models were fitted to the data. Models were fitted to test for the effects of socioeconomic status (ownership of TV, mobile phone, motorcycle, bicycle and car), sources of water for drinking, cooking and bathing, internal displacement, nutritional status, river distance and Community Directed Treatment with Ivermectin (CDTI) on the risk of NS to occur in a given household.

## Results

A total of 2,263 households in 92 villages (13 bomas) were surveyed, including 22,411 persons, of which 607 were identified as probable NS cases from 457 households. Of the persons surveyed, 58.4% (13,083/22,411) were ≤18 years of age and 50.2% were males (Table 2).

### Prevalence of NS

The overall prevalence of NS in the Greater Mundri area was 2.7% and in persons ≤ 18 years of age, 0.9%. The prevalence varied considerably across boma’s, with Diko having the highest (13.7%) and Lui the lowest (1.2%) amongst all age groups, while in persons ≤ 18 years of age, Witto and Lui/Biyokoriwa had the highest (4.5%) and lowest (0.2%) prevalence, respectively (***Table 2***). ‘New- NS’ cases, with onset of symptoms less than 12 months prior to study inclusion, represented 19% (114/607) of all cases identified, which is around 0.5% of the total population surveyed.

**Table 2:** Prevalence of nodding syndrome by Boma in Mundri East and West Counties, South Sudan.

Name of boma	Number of participants surveyed (all age groups)	Number of participants surveyed (≤ 18 years)	Number of NS cases identified (all age groups)	Number of new NS cases (≤ 18 years)	Prevalence of NS (%, all age groups)	Prevalence of NS (%, ≤ 18 years)
**Amadi**	1307	778	32	14	2.4	1.8
**Biyokoriwa**	606	469	15	1	2.5	0.2
**Delewa**	2077	1182	72	4	3.5	0.3
**Diko**	517	256	71	8	13.7	3.1
**Gulu**	458	260	16	2	3.5	0.8
**Kulundu**	805	468	18	3	2.2	0.6
**Lui**	2161	1294	27	3	1.2	0.2
**Mbara**	1069	627	17	4	1.6	0.6
**Mideh**	284	159	16	3	5.6	1.9
**Moba**	110	53	3	2	2.7	3.8
**Mundri**	11384	6555	249	28	2.2	0.4
**Witto**	481	286	19	13	4.0	4.5
**Yeri**	1152	696	52	29	4.5	4.2
Total	**22411**	**13083**	**607**	**114**	**2.7**	**0.9**

### Spatial analysis

**Fig 1:**
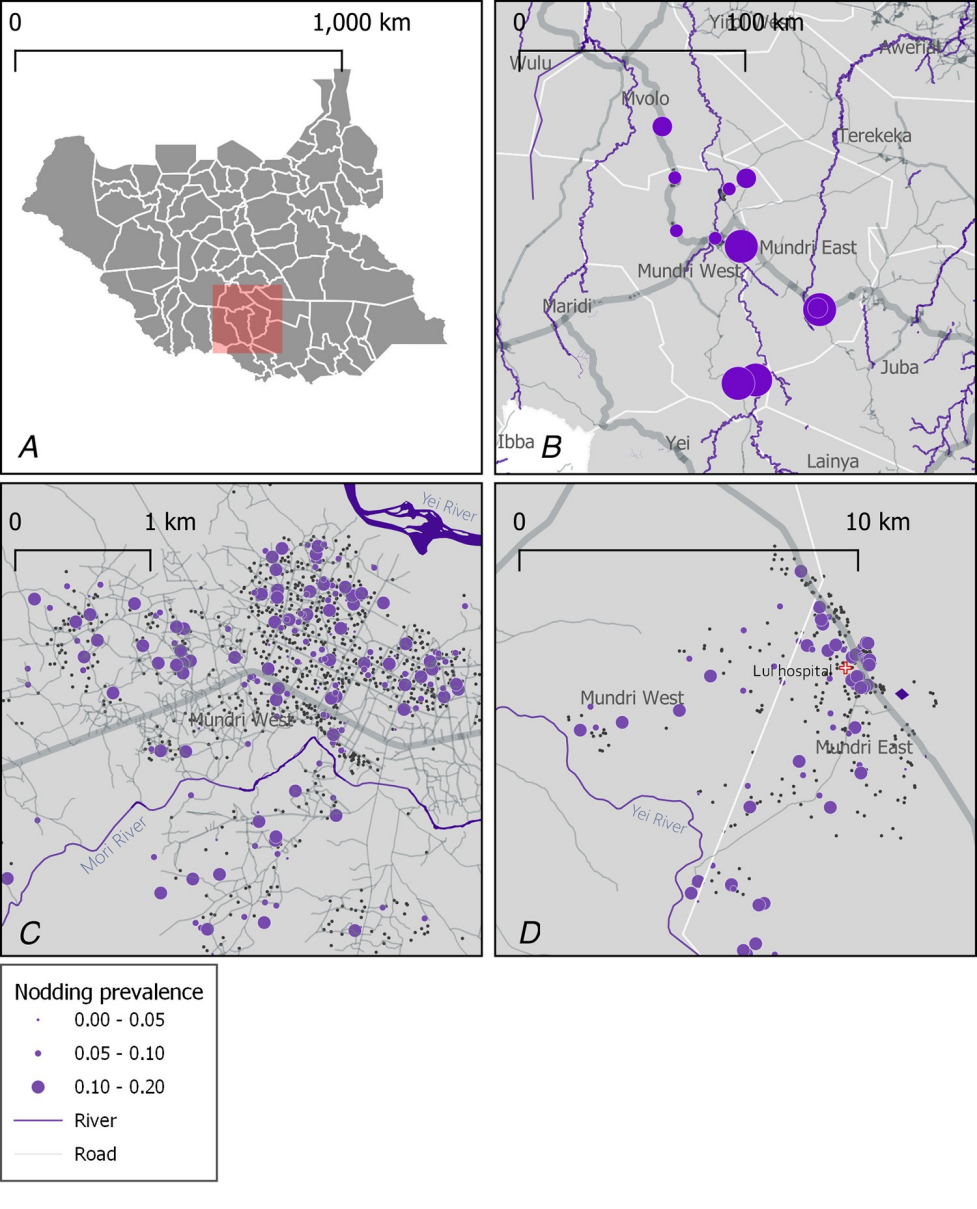
Spatial distribution of NS in the Greater Mundri Area, South Sudan. General location of the study site (A), NS prevalence across boma in the study area (B), and (C) distribution of NS affected households in Mundri (C) and Lui (D) villages. The administrative boundaries were contributed by UNOCHA South Sudan: https://data.humdata.org/organization/6d0c317d-5075-41d8-9dab-568edbb3d409.

The geographical distribution of NS is significantly clustered between adjacent households, with odds increasing to 6.9 (95% CI 4.2–11.5) if the surrounding household has an NS case. Mapping at household level showed that multiple clusters of high NS prevalence exist within the communities surveyed and concentrated within Mundri town and along the main branch of the Yei River (***Fig 1***). Although our data found few households with more than one NS case, our analysis did not show clustering of NS cases within the same family as described by previous studies.

### Risk factors for NS

The results of the univariate and multivariate analysis for the effects of socioeconomic status, sources of water for drinking, cooking and bathing, internal displacement, nutritional status, river distance and community directed treatment with Ivermectin (CDTI) on the risk of NS for a given household are shown in Table 3.

**Table 3:** Univariate and multivariate analysis of risk factors for nodding syndrome, South Sudan.

Covariates	Univariate			Multivariate		
	*OR (95% CI)*	*Z-value*	*P-value*	*OR (95% CI)*	*Z-value*	*P-value*
**CLUSTERING**						
lagNS (spatial effect)	6.9 (4.2–11.5)	7.5	0.000***	5.3 (3.2–9.1)	6.2	0.000***
**EXPOSURE TO ANIMAL**						
Cows	1.2 (0.8–1.7)	0.9	0.367			
Goats	1.1 (0.8–1.4)	0.7	0.485			
Sheep	1.3 (0.7–2.4)	0.8	0.396			
Poultry	1.4 (1.1–1.8)	2.8	0.006**	1.45 (1.13–1.88)	2.9	0.004**
**SOCIOECONOMIC STATUS**						
Television	0.6 (0.3–1.2)	-1.4	0.161			
Mobile phone	0.7 (0.5–0.9)	-3.3	0.001***	0.72 (0.57–0.92)	-2.6	0.009**
Motorcycle	0.9 (0.6–1.2)	-0.9	0.380			
Bicycle	1.0 (0.7–1.3)	-0.2	0.826			
Car	0.5 (0.1–1.3)	-1.3	0.189			
**SOURCE OF DRINKING WATER**						
Borehole	0.6 (0.4–1.0)	-2.1	0.038*			
Well	1.2 (0.3–4.5)	0.3	0.776			
Spring	4.8 (0–1.97)	0.0	0.986			
Rain	0.00	0.0	0.986			
River	1.9 (1.1–3.2)	2.5	0.014**			
Pond	0.3 (0.0–1.1)	-1.6	0.112			
Tanker	1.3(0–4.78)	0.0	0.983			
**SOURCE OF COOKING WATER**						
Borehole	0.6 (0.4–1.0)	-1.9	0.056*			
Well	0.9 (0.2–3.2)	-0.1	0.930			
Spring	1.3 (0–4.78)	0.0	0.983			
Rain	2.9 (0.3–24.1)	1.0	0.295			
River	1.8 (1.1–3.1)	2.3	0.024*			
Pond	0.4 (0.1–1.2)	-1.5	0.131			
**SOURCE OF HANDWASHING WATER**						
Borehole	0.6 (0.4–1.0)	-1.9	0.061			
Well	0.9 (0.2–3.2)	-0.1	0.930			
Spring	4.8 (0–1.97)	0.0	0.986			
Rain	1.9 (0.2–11.6)	0.7	0.484			
River	1.8 (1.1–3.0)	2.3	0.024*			
Pond	0.6 (0.2–1.3)	-1.2	0.213			
**SOURCE OF BATHING WATER**						
Borehole	0.7 (0.4–1.1)	-1.7	0.090			
Well	1.3 (0.3–4.0)	0.4	0.697			
Spring	4.8 (0–8.27)	0.0	0.984			
Rain	2.9 (0.5–15.8)	1.3	0.199			
River	1.8 (1.1–3.0)	2.2	0.028*			
Pond	0.3 (0.1–0.9)	-2.0	0.048*			
Tanker	1.0 (1.0–1.2)	1.2	0.220			
**INTERNAL DISPLACEMENT**						
Moved from study area	0.9 (0.6–1.2)	-0.9	0.383			
Lived in IDP camps	0.8 (0.4–1.3)	-0.7	0.502			
**NUTRITIONAL STATUS**						
Ate smaller meal size	1.0 (0.5–1.9)	0.1	0.923			
Ate fewer meal frequency	1.0 (0.5–1.9)	0.1	0.923			
Ate no meal	1.3 (0.9–1.8)	1.3	0.193			
Ate poor quality meal	1.1 (0.5–1.9)	0.2	0.870			
**RIVER DISTANCE**						
River distance	0.9 (0.9–1.0)	-2.5	0.014**	0.93 (0.88–0.97)	-0.3	0.002***
**CDTI**						
Ivermectin intake	1.46 (0.97–2.21)	-8.5	0.021*			

OR = Odds Ratio, CDTI = Community Directed Treatment with Ivermectin

Significance level: P-value ≤ 0.001 ***, P-value ≤ 0.01 **, P-value ≤ 0.05 *

In the univariate analysis, ownership of poultry was found to increase the risk of having NS case in households (OR 1.4, 95% CI 1.1–1.8). We also found that all behaviors around rivers, including fetching/using river water for drinking (OR 1.9, 95% CI 1.1–3.2), cooking (OR 1.8, p = 0.024), hand washing (OR 1.8, 95% CI 1.1–3.1) and bathing (OR 1.8, 95% CI 1.1–3.0) increases the risk of having NS in households. In addition, living near rivers significantly increases the risk of having NS in households (OR 0.9, 95% CI 0.9–1.0). Moreover, ownership of mobile phone was shown to significantly associate with a reduced risk of having NS case in households (OR 0.7, 95% CI 0.5–0.9). Finally, there is a very low ivermectin intake (CDTI coverage) in the Greater Mundri Area, but a significantly higher proportion of people with NS (11.9%) took ivermectin compared to those without NS (6.5%) (OR 1.5, 95% CI 0.9–2.2). No significant associations were found with other covariates, including displacement/migration and nutritional status.

Risk factors, which were significantly associated with NS in the univariate analysis were included in a multivariable model. Since covariates related to exposure to river were found to be strongly correlated (>80%), only the covariate describing distance to a river was retained in the multivariable regression model (Table 3). Ownership of poultry within the household premises (OR 1.45, 95% CI 1.13–1.88) and living near a river (OR 0.9, 95% CI 0.88–0.97) still increased the risk for NS, while ownership of a mobile phone remained protective of having NS in households (OR 0.7, 955 CI 0.57–0.92).

## Discussion

To our knowledge, this is the largest population-based study to determine the prevalence and the epidemiological risk factors of nodding syndrome to date. The high prevalence of NS observed across the communities in the Greater Mundri area are in line with previous reports from smaller studies in restricted areas [2,7]. The observed prevalence rate is also in agreement with previous reports that South Sudan has foci with the highest prevalence of NS cases compared to other NS-affected countries [5,6,10,12,18]. Our study observed higher prevalence of NS amongst those ≥18 years compared to those ≤18 years of age. The possible explanation for this may be that the prevalence of NS accumulates as the morbidities do not disappear and mortality is relatively low. Furthermore, it could also suggest that the incidence of NS is dropping over the years while the existing cases grow into adulthood. Yet, the relatively high number of new-onset cases suggests that new cases are continuing to emerge in South Sudan, which calls for urgent interventions that may reduce the incidence of this overwhelming disease.

The results of the spatial analysis confirmed previous observations that NS cases are geographically clustered within communities. The presence of NS in these communities may therefore be a result of a common ecological exposure to either chemical or a biological agent. However, the environmental factors which were found to be significantly associated to NS in the multivariate model did not fully account for the spatial clustering observed. The possible explanations for this finding are (1) the ecological risks to which children were found to be subjected and which were included in the model, affect households in a wider geographic area. In such case, the presence of a risk factor at a single household would increase the risk of NS in surrounding household, for example due to local mobility of individuals or due to the presence of a vector and (2) additional environmental factors, which coincide with the observed patterns but which were not included in the model drive the strong spatial clustering.

Our study is the first to observe that ownership of poultry increases the risk of having an NS case in a household. This novel finding suggests a possible zoonotic risk factor. Of the known poultry diseases that can potentially transmit to humans including salmonella, campylobacter, histoplasmosis or avian influenza, none are known to cause a neurodegenerative disease. However, poultry could be a reservoir of a yet to be identified neurotropic virus or other infectious agent that may lead to NS. There is need to further evaluate this novel link.

Households which reported to own a mobile phone were found to have a lower risk of NS. Since ownership of a mobile phone may be an indication of wealth, this finding may be in line with previous reports that NS occurs amongst families of low socioeconomic status or impoverished [8,19]. However, we found no association with poverty and history of displacement. Families of low socioeconomic status may be less educated and confined to their rural villages of origin near the rivers and therefore highly exposed to the NS triggering factor related to the use of river water or life near the rivers than those of a better socioeconomic status.

The strong association between NS and all activities involving river water and living near the river is in line with previous findings that NS cases clusters around fast-flowing rivers, which are infested with the blackflies and therefore the potential link between NS and OV [8,11,20,21]. This is also in line with previous studies of onchocerciasis-associated epilepsies that included cases of both epilepsy and NS in Maridi and Mvolo Counties of Western Equatoria State in South Sudan. These studies reported higher rates of epilepsy and NS in villages in Maridi County that are located near the Maridi River, close to the Maridi Dam, the blackfly breeding site in the area [20,22]. Similarly, in Mvolo, higher rates were reported in villages near the Naam River; also, a blackfly bleeding site [23]. However, these findings need to be further explored for other epidemiological and environmental risk factors around the rivers, which are not necessarily related to OV. Although association with CDTI and larviciding of rivers have been suggested to respectively reduce the cumulative incidence of NS and epilepsy between 2012–2017 and 1994–2018 in northern Uganda and Western Uganda [24,25], other factors, which are also related to living near the rivers and/or using river water; such as a neurotropic virus transmitted by the blackflies or contaminated rivers with toxins like heavy metals or organic substances needs to be further evaluated.

Our study has some limitations: the challenges of recall related to medical history including the onset of NS symptoms; limited access to some NS affected communities due to security issues or poor road network; armed conflict that resulted into movement of the population in and out of the study area and non-response due to stigma could have influenced the results; and the identification of NS cases was mainly made by clinical officer, nurse and medical doctor but not confirmed by a neurologist. Despite the limitations, we consider our data to be a representative sample of sufficient size to estimate the prevalence, detect spatial patterns due to good geographical coverage, and sufficiently powered for inferential analysis. Also, all the NS cases identified were based on a pre-defined criterion, which was adopted from the 2012 nodding syndrome case definition (International Scientific Meeting on Nodding Syndrome in Kampala, Uganda).

In conclusion, our study documented high prevalence of NS and provided evidence that newer cases continue to arise in the Greater Mundri area of South Sudan. It has also documented newer association of NS with poultry and strongly confirmed previous observation that NS occurs around fast-flowing rivers. There is urgent need to further investigate other unidentified factors that are related to the use of river water and/or living near rivers than previously studied. In addition, there is need to further explore the unexpected NS link with poultry. Identifying the possible risk factor of NS will provide the basis for preventive interventions that may reduce the growing incidence of the disease. Lastly, public health interventions such as CDTI and larviciding of the rivers, and “slash and clear” of blackflies breeding sites that were respectively reported to have effectively reduce the incidence of NS and blackflies biting rates should be further evaluated using randomized controlled trials while scientists continue to investigate the definitive etiological and risk factors of NS [24,26].

## Supporting information

S1 Table Total numbers and percentages. (XLSX)

S1 Questionnaire used for the study Household survey. (XLS)

## References

[1] Spencer PS, Palmer VS, Jilek-Aall L: Nodding syndrome: origins and natural history of a longstanding epileptic disorder in sub-Saharan Africa. *African Health Sciences* 2013, 13(2):176–182. doi: 10.4314/ahs.v13i2.1 24235914 PMC3824511

[2] World Health Organization. Nodding syndrome. Geneva: WHO, 2018. http://www.who.int/onchocerciasis/symptoms/nodding_syndrome/en/ (Accessed: 5 December 2018)

[3] Waals FWvd, Goudsmit J, Gajdusek DC: Characteristics of Highly Prevalent Seizure Disorders in the Gbawein and Wroughbarh Clan Region of Grand Bassa County, Liberia. *Neuroepidemiology* 1983, 1983(2):35–44.

[4] Lenaerts E, Mandro M, Mukendi D, Suykerbuyk P, Dolo H, Wonya’Rossi D, Ngave F, Ensoy-Musoro C, Laudisoit A, Hotterbeekx A et al: High prevalence of epilepsy in onchocerciasis endemic health areas in Democratic Republic of the Congo. *Infectious Diseases of Poverty* 2018, 7(68). doi: 10.1186/s40249-018-0452-1 30064504 PMC6069757

[5] Siewe JFN, Ngarka L, Tatah G, Mengnjo MK, Nfor LN, Chokote ES, Boullé C, Nkouonlack C, Dema F, Nkoro GA et al: Clinical presentations of onchocerciasis-associated epilepsy (OAE) in Cameroon. *Epilepsy Behav* 2019, 90(2019):70–78. doi: 10.1016/j.yebeh.2018.11.008 30513438

[6] Metanmo S, Boumediene F, Preux PM, Colebunders R, Siewe Fodjo JN, de Smet E, Yangatimbi E, Winkler AS, Mbelesso P, Ajzenberg D: First description of Nodding Syndrome in the Central African Republic. *PLoS Negl Trop Dis* 2021, 15(6):e0009430. doi: 10.1371/journal.pntd.0009430 34143783 PMC8244846

[7] Tumwine JK, Vandemaele K, Chungong S, Richer M, Anker M, Ayana Y, Opoka ML, Klaucke DN, Quarello A, Spencer PS: Clinical and epidemiologic characteristics of nodding syndrome in Mundri County, southern Sudan. *African Health Sciences* 2012, 12(3):242–248. doi: 10.4314/ahs.v12i3.1 23382736 PMC3557692

[8] Spencer PS, Vandemaele K, Richer M, Palmer VS, Chungong S, Anker M, Ayana Y, Opoka ML, Klaucke BN, Quarello A et al: Nodding syndrome in Mundri county, South Sudan: environmental, nutritional and infectious factors. *African Health Science**s* 2013, 13(2):183–204. doi: 10.4314/ahs.v13i2.2 24235915 PMC3824482

[9] Sejvar JJ, Kakooza AM, Foltz JL, Makumbi I, Atai-Omoruto AD, Malimbo M, Ndyomugyenyi R, Alexander LN, Abang B, Downing RG et al: Clinical, neurological, and electrophysiological features of nodding syndrome in Kitgum, Uganda: an observational case series. *Lancet neurol* 2013, 12(2):166–174. doi: 10.1016/S1474-4422(12)70321-6 23305742

[10] Mukendi D, Tepage F, Akonda I, Siewe JNF, Rotsaert A, Ndibmun CN, Laudisoit A, Couvreur S, Kabutako B, Menon S et al: High prevalence of epilepsy in an onchocerciasis endemic health zone in the Democratic Republic of the Congo, despite 14 years of community-directed treatment with ivermectin: A mixed-method assessment. *International Journal of Infectious Diseases* 2019, 79:187–194. doi: 10.1016/j.ijid.2018.10.021 30711145 PMC6353816

[11] Kitara DL, Oh J, Mwaka AD: Nodding Syndrome in Uganda—A Disease Cluster: An Epidemiological Dilemma. *Pacific Journal of Medical Sciences* 2013:21–33.

[12] Iyengar PJ, Wamala J, Ratto J, Blanton C, Malimbo M, Lukwago L, Becknell S, Downing R, Bunga S, Sejvar J et al: Prevalence of nodding syndrome—Uganda, 2012–2013. *CDC MMWR—Morbidity & Mortality Weekly Report* 2014, 63(28):603–606. 25029112 PMC5779414

[13] Winkler AS, Friedrich K, Konig R, Meindl M, Helbok R, Unterberger I, Gotwald T, Dharsee J, Velicheti S, Kidunda A et al: The head nodding syndrome—clinical classification and possible causes. *Epilepsia* 2008, 49(12):2008–2015. doi: 10.1111/j.1528-1167.2008.01671.x 18503562

[14] de Polo G, Romaniello R, Otim A, Benjamin K, Bonanni P, Borgatti R: Neurophysiological and clinical findings on Nodding Syndrome in 21 South Sudanese children and a review of the literature. *Seizure* 2015, 31:64–71. doi: 10.1016/j.seizure.2015.07.006 26362379

[15] Conflict Sensitivity Resource Facility. Demography. Mundri West County, Western Equatoria State. South Sudan: CSRF, 2020. https://www.csrf-southsudan.org/county_profile/mundri-west/ (Accessed: 2 July 2021)

[16] Conflict Sensitivity Resource Facility. Demography. Mundri East, Western Equatoria State. South Sudan: CSRF, 2020. https://www.csrf-southsudan.org/county_profile/mundri-east/ (Accessed: 2 July 2021)

[17] WHO: International Scientific Meeting on Nodding Syndrome Kampala, Uganda. In. Geneva, Switzerland; 2012: 1–42. https://www.who.int/neglected_diseases/diseases/Nodding_syndrom_Kampala_Report_2012.pdf

[18] Mmbando BP, Suykerbuyk P, Mnacho M, Kakorozya A, Matuja W, Hendy A, Greter H, Makunde WH, Colebunders R: High prevalence of epilepsy in two rural onchocerciasis endemic villages in the Mahenge area, Tanzania, after 20 years of community directed treatment with ivermectin. *Infectious Diseases of Poverty* 2018, 7(1):64. doi: 10.1186/s40249-018-0450-3 29921319 PMC6009039

[19] Dowell SF, Sejvar JJ, Riek L, Vandemaele KA, Lamunu M, Kuesel AC, Schmutzhard E, Matuja W, Bunga S, Foltz J et al: Nodding syndrome. *Emerg Infect Dis* 2013, 19(9):1374–1384. doi: 10.3201/eid1909.130401 23965548 PMC3810928

[20] Colebunders R J YC, Olore PC, Puok K, Bhattacharyya S, Menon S, Abd-Elfarag G, Ojok M, Ensoy-Musoro C, Lako R et al: High prevalence of onchocerciasis-associated epilepsy in villages in Maridi County, Republic of South Sudan: A community-based survey. *Seizure* 2018, 63:93–101. doi: 10.1016/j.seizure.2018.11.004 30468964 PMC6291739

[21] Kaiser C, Asaba G, Rubaale T, Tukesiga E, Kipp W: Onchocerciasis-Associated Epilepsy with Head Nodding Seizures-Nodding Syndrome: A Case Series of 15 Patients from Western Uganda, 1994. *Am J Trop Med Hyg* 2018, 99(5):1211–1218. 30226148 10.4269/ajtmh.18-0511 PMC6221207

[22] Lakwo TL, Raimon S, Tionga M, Siewe Fodjo JN, Alinda P, Sebit WJ, Carter JY, Colebunders R: The Role of the Maridi Dam in Causing an Onchocerciasis-Associated Epilepsy Epidemic in Maridi, South Sudan: An Epidemiological, Sociological, and Entomological Study. *Pathogens* 2020, 9(4):24. 10.3390/pathogens9040315 PMC7238195 32344586

[23] Raimon S, Dusabimana A, Abd-Elfarag G, Okaro S, Carter JY, Newton CR, Logora MY, Colebunders R: High Prevalence of Epilepsy in an Onchocerciasis-Endemic Area in Mvolo County, South Sudan: A Door-To-Door Survey. *Pathogens* 2021, 10(5):14. 10.3390/pathogens10050599 PMC8157079 34068976

[24] Gumisiriza N, Mubiru F, Siewe Fodjo JN, Mbonye Kayitale M, Hotterbeekx A, Idro R, Makumbi I, Lakwo T, Opar B, Kaducu J et al: Prevalence and incidence of nodding syndrome and other forms of epilepsy in onchocerciasis-endemic areas in northern Uganda after the implementation of onchocerciasis control measures. *Infectious Diseases of Poverty* 2020, 9(1):12. doi: 10.1186/s40249-020-0628-3 32114979 PMC7050130

[25] Gumisiriza N., et al., *Changes in epilepsy burden after onchocerciasis elimination in a hyperendemic focus of western Uganda*: *a comparison of two population-based*, *cross-sectional studies*. Lancet Infect Dis., 2020. 20(11): p. 1315–1323. doi: 10.1016/S1473-3099(20)30122-5 32598869

[26] Raimon S., et al. (2021). "“Slash and Clear”, a Community-Based Vector Control Method to Reduce Onchocerciasis Transmission by Simulium sirbanum in Maridi, South Sudan: A Prospective Study." Pathogens 10(1329). 10.3390/pathogens10101329 PMC8538802 34684277

